# COBRAxy: constraint-based metabolic modeling in Galaxy

**DOI:** 10.1093/bioinformatics/btaf670

**Published:** 2025-12-19

**Authors:** Francesco Lapi, Luca Milazzo, Lihao Lin, Isabella Cecilia Rizzo, Bruno Giovanni Galuzzi, Chiara Damiani

**Affiliations:** Department of Biotechnology and Biosciences, University of Milano-Bicocca, Milan, 20126, Italy; Laboratory of Computational Systems Biotechnology, Ecole polytechnique fédérale de Lausanne (EPFL), Lausanne, 1015, Switzerland; Department of Biotechnology and Biosciences, University of Milano-Bicocca, Milan, 20126, Italy; Department of Biotechnology and Biosciences, University of Milano-Bicocca, Milan, 20126, Italy; Institute of Bioimaging and Complex Biological Systems (IBSBC), National Research Council (CNR), Via F.lli Cervi, 93, Segrate, 20054, Italy; National Biodiversity Future Center (NBFC), Palermo, 90133, Italy; SYSBIO Centre of Systems Biology ISBE-IT, Milan, 20126, Italy; Department of Biotechnology and Biosciences, University of Milano-Bicocca, Milan, 20126, Italy; SYSBIO Centre of Systems Biology ISBE-IT, Milan, 20126, Italy

## Abstract

**Motivation:**

Metabolic network modeling is essential for understanding metabolic shifts occurring in complex physio-pathological processes. Currently, constraint-based modeling frameworks for metabolic networks primarily rely on Python or MATLAB libraries, requiring some coding skills. In contrast, more user-friendly tools lack essential features such as flux sampling or transcriptomic data integration.

**Results:**

We introduce COBRAxy, a Python-based tool suite integrated into the Galaxy Project. COBRAxy enables constraint-based modeling and sampling techniques, allowing users to compute metabolic flux distributions for multiple biological samples. The tool also enables the integration of medium composition information to refine flux predictions. Additionally, COBRAxy provides a user-friendly interface for visualizing significant flux differences between populations on an enriched metabolic map. This extension provides a comprehensive and accessible framework for advanced metabolic analysis, enabling researchers without extensive programming expertise to explore complex metabolic processes.

**Availability and implementation:**

COBRAxy is available in the Galaxy ToolShed https://toolshed.g2.bx.psu.edu/view/bimib/cobraxy/9f78303dbd88.

## 1 Introduction

Metabolism is a complex, dynamic system regulated at multiple levels, where enzyme activity, substrate availability, and interdependent pathways create non-linear interactions that -omics data alone cannot fully capture. Understanding metabolic shifts in health and disease requires tools that integrate multi-omics data and provide accessible insights, even for researchers without programming expertise.

However, there is a lack of accessible computational frameworks that enable researchers, particularly those without a strong programming background, to apply advanced techniques such as constraint-based modeling and sampling. State of the art libraries, such as COBRApy (Ebrahim *et al.* 2013) and COBRA Toolbox (Heirendt *et al.* 2019), require programming skills, which often limits their use in studying complex metabolic shifts in health and disease. On the contrary GUI-based tools such as Escher (King *et al.* 2015), Escher-FBA (Rowe *et al.* 2018), or FAME (Boele *et al.* 2012) do not enable integration of omics data into the constraint-based problem and do not support flux sampling, which is emerging as a powerful alternative to optimization methods (Herrmann *et al.* 2019). Other comprehensive and user-friendly solutions, such as CellNetAnalyzer (von Kamp *et al.* 2017) and CNApy (Thiele *et al.* 2022), offer extended functionality, but they also present certain limitations: the former requires a MATLAB installation and does not perform flux sampling, while the latter depends on Python and similarly lacks flux sampling capabilities.

In this context, the web-based bioinformatics platform Galaxy stands out as an ideal solution, promoting reproducible workflows and collaboration (The Galaxy Community 2024). Early efforts like MaREA (Graudenzi *et al.* 2018) and MaREA 2.0 (Ferrari *et al.* 2024) have leveraged Galaxy for metabolic studies, but accessible tools for constraint-based modeling and sampling remain scarce.

**Figure 1.**
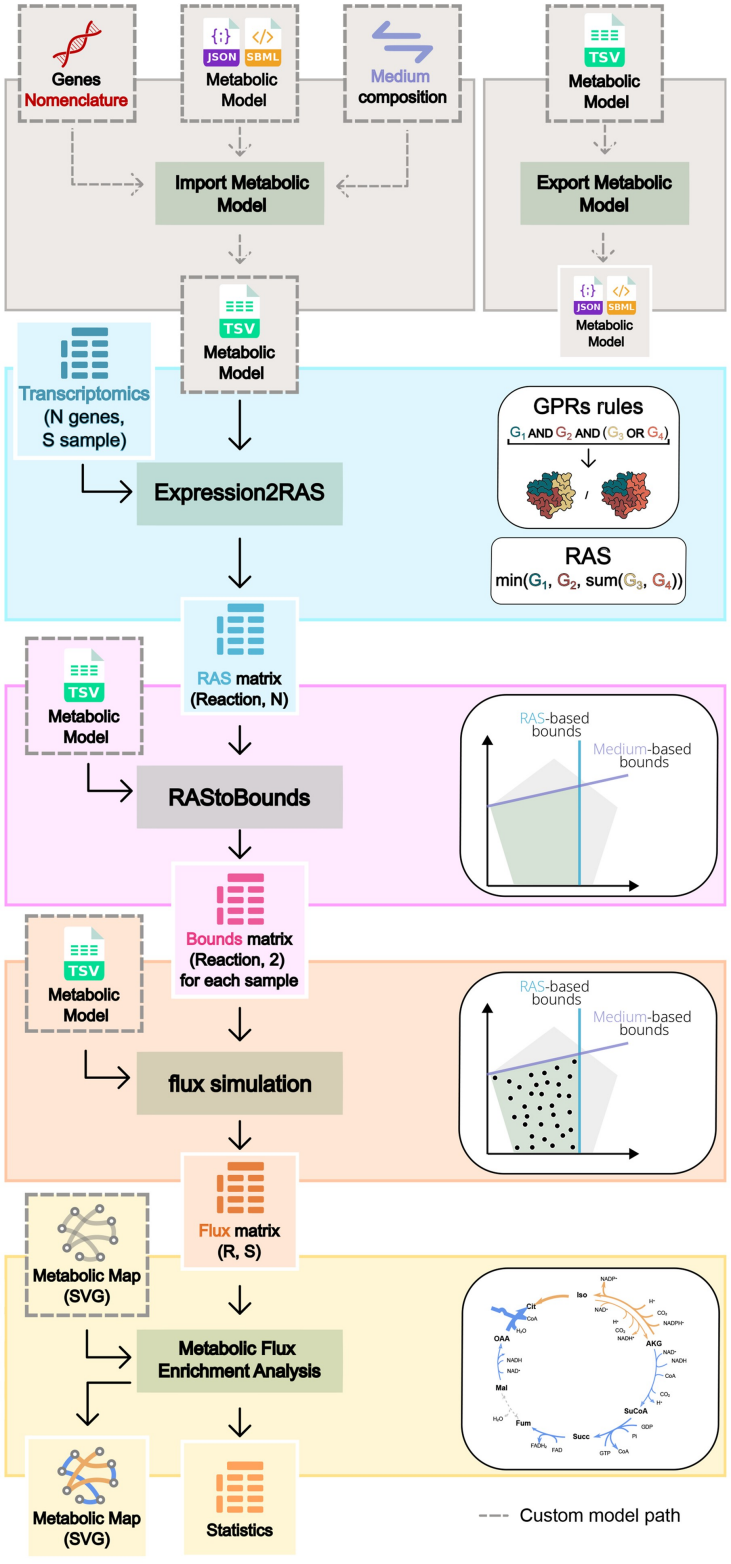
COBRAxy graphical abstract.

To address this need, we introduce the tool suite COBRAxy (Fig. 1). COBRAxy enables the integration of transcriptomics data with COBRA-based metabolic models, offering a comprehensive framework for studying metabolism in both health and disease. With COBRAxy, users can load and enrich metabolic models by incorporating transcriptomic data and adjusting the model’s medium conditions.

Using flux sampling, COBRAxy enables the estimation of metabolic flux distributions, offering valuable insights into reaction activity and flux patterns. Additionally, users can visualize statistically significant flux differences between populations on an enriched metabolic map.

By offering an intuitive and accessible platform for multi-omics integration and metabolic analysis, COBRAxy meets the growing need for tools that help researchers explore complex metabolic processes with ease.

## 2 COBRAxy

The first step, model preparation, allows users to upload genome-scale or context-specific metabolic models. With the tool *Import Metabolic Model*, users can load their own metabolic model and extract relevant information into a tabular file containing all the main data for further analysis, including gene-protein-reaction (GPR) rules, reaction bounds, and medium composition. The tool accepts an input file describing a metabolic network, including reactions, metabolites, genes, and bounds, for flux simulation. It supports the main formats commonly used for metabolic models, namely JSON (.json), SBML (.xml), MATLAB (.mat), and YAML (.yaml), as well as compressed versions (.zip, .gz, and.bz2). Notably, users should be aware that SBML files generated by MATLAB or other tools may not always be fully compatible with the *Import Metabolic Model* tool, which is based on COBRApy.

Pre-existing models, such as ENGRO2 (Di Filippo *et al.* 2022), or Recon3D (Brunk *et al.* 2018), can be used directly, bypassing the need to load a custom model, as they are already prepared for use in the subsequent tools. For completeness, we have also introduced the *Export Metabolic Model* tool, which allows users to export metabolic models from the tabular format into a standard model file format.

The next step involves quantifying the potential activity of the reactions in the metabolic model. The user can upload tabular data related to two or more transcriptomics profiles (single-cell or bulk) and map each gene expression profile to the corresponding metabolic model. This is achieved through Reaction Activity Scores (RAS) (Graudenzi *et al.* 2018), which are computed using the *Expression2RAS* tool. These scores are derived from gene expression data by leveraging the GPR rules embedded in the model. GPR rules link genes to reactions through Boolean expressions, where “OR” is interpreted as a sum (indicating redundancy) and “AND” as a minimum (indicating co-dependency). For each reaction, the RAS reflects the expression levels of its associated genes, as defined by these rules. The calculated RAS values are then normalized within the *RAStoBounds* tool. Normalization is performed by dividing each RAS value by the maximum RAS across all samples, ensuring that scores fall within a comparable range. These normalized RAS values are subsequently used to generate dynamic reaction bounds for the metabolic model. Specifically, the RAS values act as scaling factors to adjust the upper and lower bounds of each reaction, linking the model’s constraints directly to the underlying transcriptomics data. If a custom growth medium is specified, it can further refine the bounds by limiting exchange reactions based on the availability of extracellular metabolites.

Once set, these bounds define the model’s constraints, enabling exploration of the omics-customized region of feasible flux distributions. Additionally, users can export the transcriptionally derived metabolic models generated by RAStoBounds.

The *Flux Simulation* tool supports different state-of-the-art techniques to explore this region. They include optimization-based techniques, such as parsimonious-FBA (Lewis *et al.* 2010), Flux Variability Analysis (FVA) (Mahadevan and Schilling 2003), and sensitivity analysis via single reaction deletion, as well as flux sampling. Unlike optimization-based techniques, which provide a single optimal flux distribution, flux sampling generates multiple feasible solutions. This probabilistic approach captures flux variability and highlights alternative pathways, making it especially useful for studying complex metabolic systems. The tool can generate flux samples that satisfy the steady-state condition, meaning the system is balanced, using Corner-Based Sampling (CBS) (Galuzzi *et al.* 2024) or Improved Artificial Centering Hit-and-Run sampler (OPTGP) (Megchelenbrink *et al.* 2014). The number of flux samples is a customizable parameter: the user can specify how many flux solutions to sample and have the option to partition the sampled flux distribution into distinct batches. The tool provides summary statistics like mean, median, and quantiles for the sampled fluxes.

The final step uses the *Metabolic Flux Enrichment Analysis* tool to compare fluxes between two or more groups of samples. Users can provide the input in the form of a separated dataset of fluxes for each group or a unique dataset plus a file assigning each sample to a group. The tool supports three types of comparisons: comparing all possible pairs of groups (1 versus 1), comparing one group against all the others (1 versus All), or comparing each group individually against a control group (1 versus Control). Users can also select a metabolic map to visualize the results. This map must be consistent with the metabolic model and can be uploaded by the user.

The method performs a user-defined statistical test (e.g., the Kolmogorov–Smirnov test) to identify statistically significant differences, and calculates the fold change according to Equation (1):


(1) $$\frac{avg_{1}-avg_{2}}{\left|avg_{1}\right|+\left|avg_{2}\right|},$$

where $avg_{1}$ and $avg_{2}$ are the average values of all the fluxes across the samples in the two comparison groups. By default, the *P*-value threshold is set to 0.05 and the fold change threshold to 1.2 (at least 20% difference), but these values can be adjusted by the user. Only reactions that pass both tests will be highlighted in the output map. The tool generates comparison maps based on the selected method. Arrows are colored sky blue or orange depending on whether the first group is down- or upregulated compared to the second one. If the flux values have opposite signs between two groups, indicating a different direction of usage of a reversible reaction, the corresponding arrow will be highlighted in red or blue. In addition, a map is provided for each group showing the average and median flux distributions. The color map is customizable by the user. In all maps, the arrow size varies: in the comparison maps, it is based on the fold change; in the average/median maps, it reflects the flux intensity.

A table is also provided for each comparison, containing the following values for each reaction: id, *P*-value, fold change [Equation (1)], *z*-score, average of the first group, and average of the second group.

Finally, the workflow enables seamless integration of results by exporting flux distributions, statistical analyses, and visualizations in various formats. The output is compatible with clustering tools, facilitating downstream analysis and interpretation.

## 3 Case study

To demonstrate the applicability of COBRAxy, we analyzed the TCGA‐BRCA breast cancer dataset released in (Ciriello *et al.* 2015), comprising RNA Seq V2 RSEM expression profiles of biopsies from 817 patients, with matched normal tissue profiles available for 112 of them, covering 20 440 genes.

The same dataset was previously used for data mapping with MaREA (Graudenzi *et al.* 2018), indicating a consistent upregulation of reactions in the glycolytic pathway but a less clear picture of mitochondrial pathways.

Using COBRAxy, we now integrated it into the constraints of the ENGRO2 metabolic model, which encompasses 395 metabolites, 469 reactions, and 498 genes.

The step-by-step tutorial of the analysis is reported in File 1, available as supplementary data at *Bioinformatics* online. The resulting map, reported as File 2, available as supplementary data at *Bioinformatics* online, provides a much more informative portray of the distinct metabolic programs of cancer and healthy cells.

Specifically, in cancer cells, the glycolytic flux and lactate production are significantly increased, consistent with the Warburg effect, indicating a shift toward aerobic glycolysis to meet the energy and anabolic demands of the tumor. The TCA cycle displays a non-canonical behavior in cancer cells, in line with experimental results reported in Arnold *et al.* (2022). Rather than functioning as a complete cycle, some reactions carry a sustained flux to support the biosynthesis of nucleotides, amino acids, and lipids, while others are reduced, reflecting a metabolic rewiring that aligns with the specific needs of tumor cells.

Additionally, the nucleotide and lipid biosynthesis pathways carry higher flux in cancer cells, underscoring their increased demand for these processes to support growth and proliferation.

This case study highlights the metabolic shifts that differentiate cancer cells from healthy cells and underscores the utility of COBRAxy in exploring complex metabolic systems.

## 4 Conclusion

The modular design and Galaxy integration of COBRAxy make it a versatile tool suite for investigating metabolic shifts in health, disease, and diverse environmental contexts. By integrating multi-omics data with constraint-based metabolic models, it allows for detailed insights into metabolic shifts. The case study demonstrated the tool’s ability to reveal distinct metabolic patterns between cancer and healthy cells, including altered glycolytic activity, non-canonical TCA cycle behavior, and upregulated biomass production pathways. These findings underscore the utility of COBRAxy in characterizing metabolic differences, offering a robust and accessible framework for researchers to explore and interpret metabolic dynamics in diverse biological contexts.

## Supplementary Material

btaf670_Supplementary_Data

## Data Availability

The code is stored in the Galaxy ToolShed, at https://toolshed.g2.bx.psu.edu/repos/bimib/marea, and in the GitHub repository, at https://github.com/CompBtBs/COBRAxy/tree/main. A demo of the tool is available at: http://marea4galaxy.cloud.ba.infn.it/galaxy/root/login? redirect=%2Fgalaxy%2F. The case study can be reproduced by using the workflow at this link: http://marea4galaxy.cloud.ba.infn.it/galaxy/published/workflow? id=16e792953f5b45db The data for the case study are available in the GitHub repository in a zip folder called data_tutorial. In addition, we also provided a collection of more general workflows illustrating different applications of the tool at the following public Galaxy repository: http://marea4galaxy.cloud.ba.infn.it/galaxy/workflows/list_published. A detailed explanation of each workflow is available in https://github.com/CompBtBs/COBRAxy/tree/main/docs/tutorials.
