# Supplementary material for: COBRAxy: constraint-based metabolic modeling in Galaxy
Source: Bioinformatics. 2025 Dec 19;42(2):btaf670. doi: 10.1093/bioinformatics/btaf670 (PMC12926780; doi:10.1093/bioinformatics/btaf670)
Supplement: btaf670_Supplementary_Data [file btaf670_supplementary_data.zip › 11-Feb-2026_120523_SupplementaryMaterials.pdf]

Supplementary Materials

1 Tutorial

This section provides a brief tutorial to reproduce the case study presented in this article with COBRAxy. The overall workflow is depicted in Figure1 and is available as public worflow at the following link: http://marea4galaxy.cloud.ba.infn.it/galaxy/published/workflow?id=a64417ff266b740e.
A brief description and illustration of the practical usage of each tool is reported in the following.

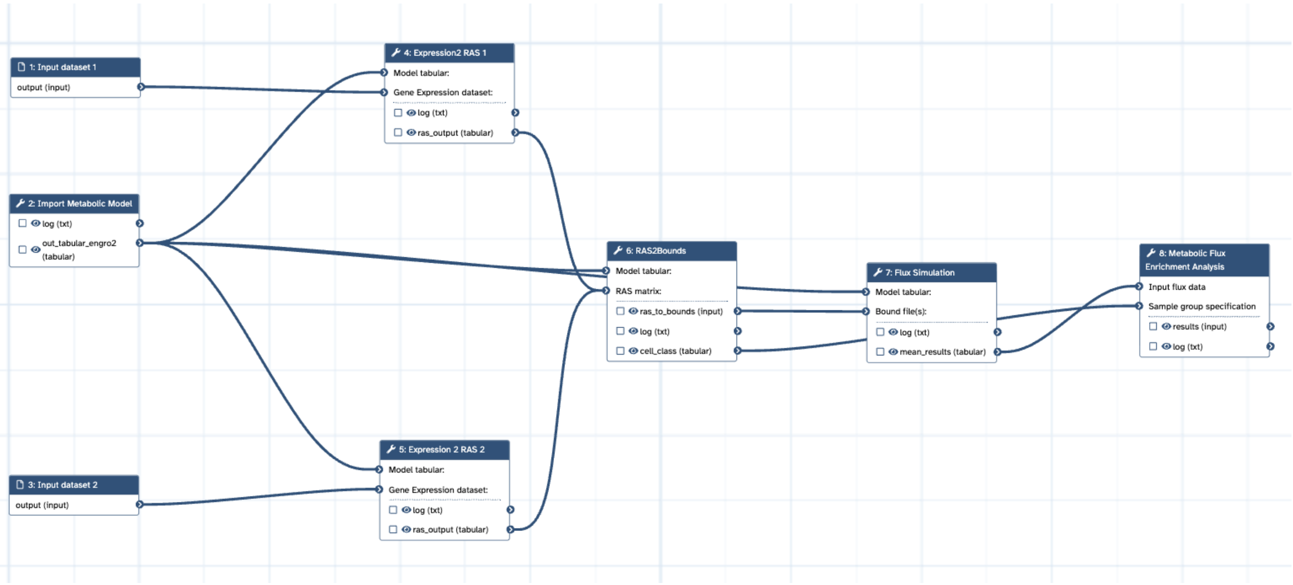

Fig. 1. Case study workflow.

Import Metabolic Model tool

This tool generates a tabular file (.tabular) containing the main information of a metabolic model, starting from either a pre-built (ENGRO2 or Recon3D) or a custom model. This tool requires three input parameters: a COBRA metabolic model (including reactions, metabolites, and genes), the growth medium (chosen from commonly used cell culture media), and the gene nomenclature format. The output consists of a .tabular file containing reaction IDs, formulas, GPR rules, reaction bounds, objective function coefficients, pathways, and medium-related reactions.

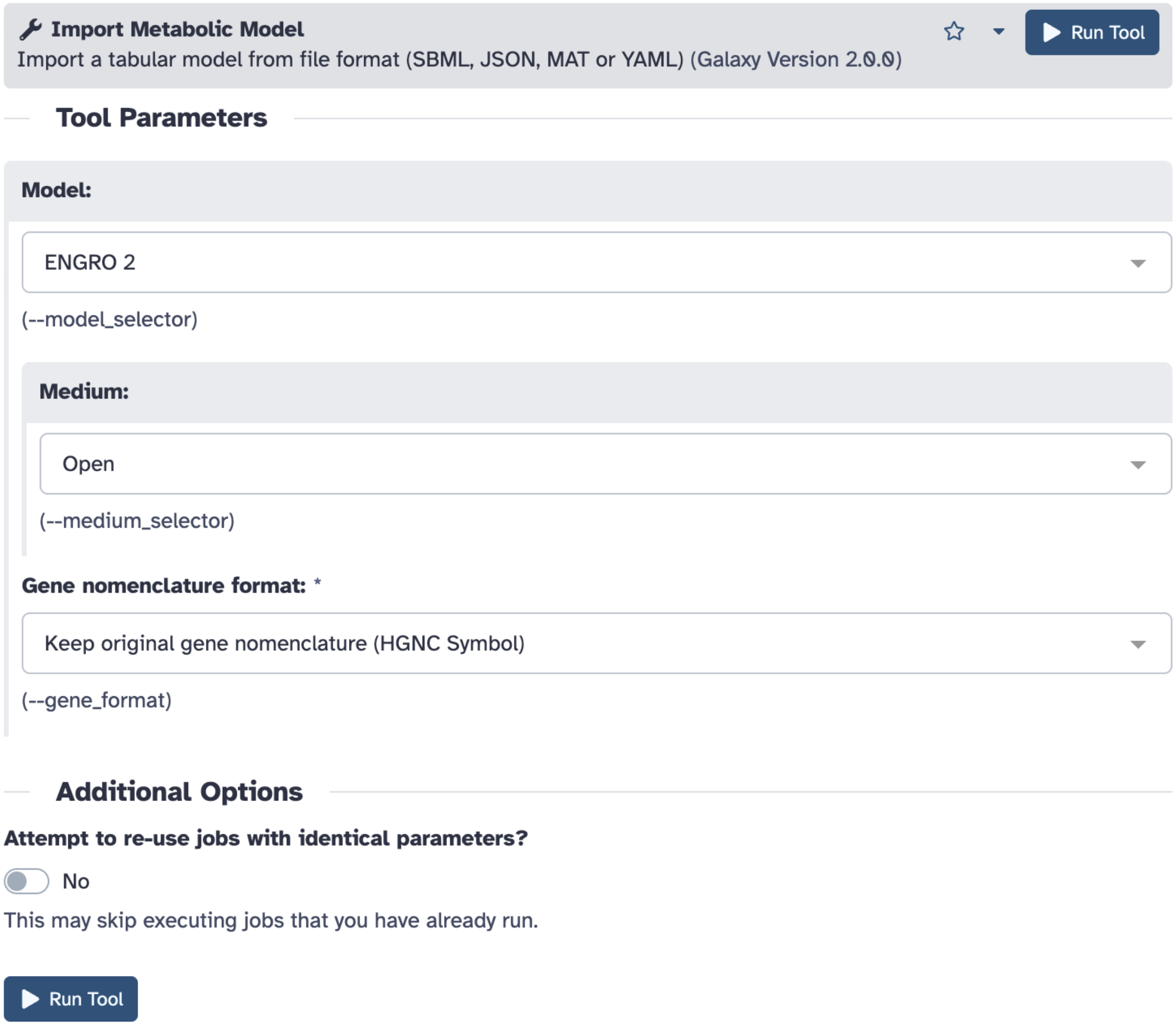

Fig. 2. Import Metabolic Model tool

Expression2RAS tool

This tool calculates the Reaction Activity Scores (RAS) based on previously defined Gene-Protein-Reaction (GPR) rules and transcriptomic data. Users must provide custom GPR rules or select predefined rules, extracted from a metabolic model using the *Import Metabolic Model* tool.
In the example shown in Figure 3, the rules from the ENGRO2 model are used. Users can also specify a name for the output dataset containing the computed RAS values.

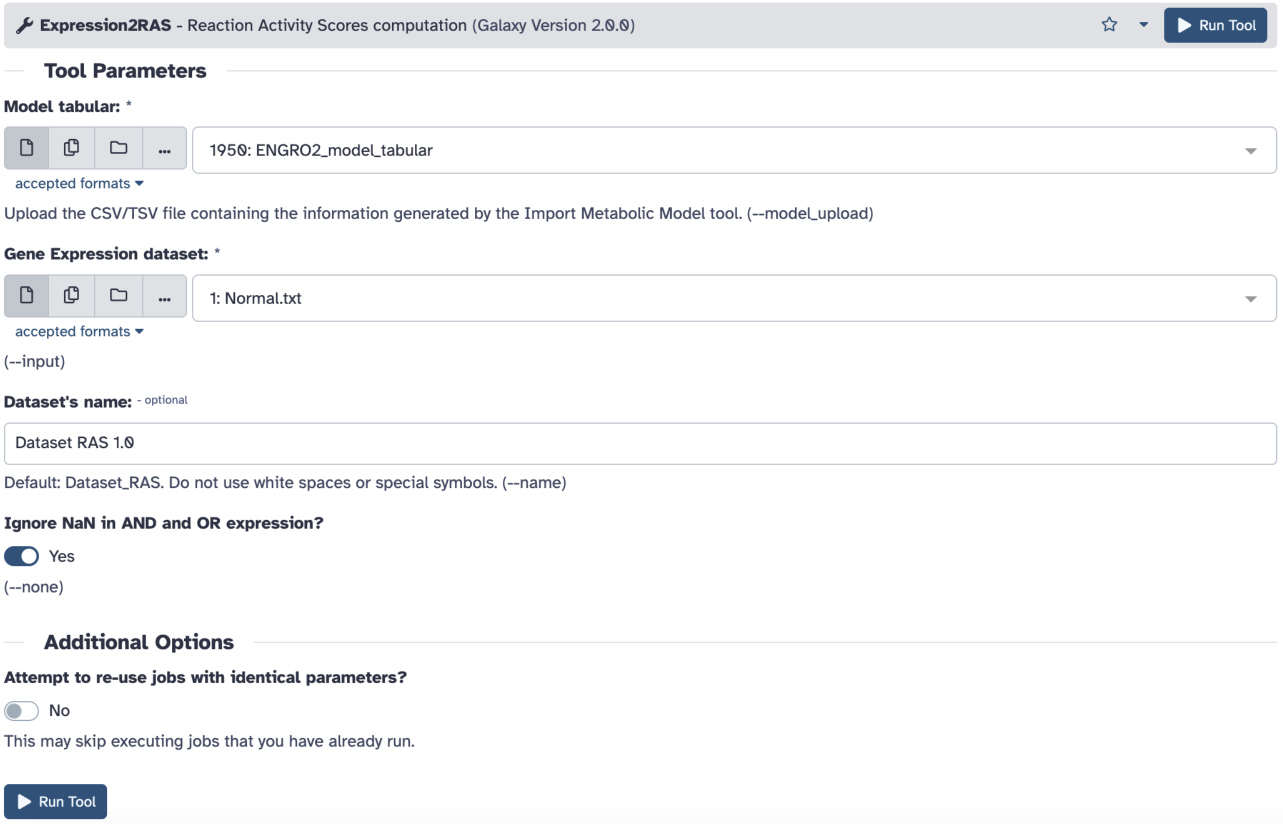

Fig. 3. Expression2RAS tool

RAStoBounds tool

After obtaining the RAS values, users can use the *RAStoBounds* tool to calculate the reaction bounds (upper and lower limits) for each metabolic model associated with each sample.
The tool generates a collection of tabular files: one per sample, containing the lower and upper bounds for all reactions. The transcriptionally-informed models created by COBRAxy can be also exported.
In the example shown in Figure 4, the ENGRO2 model is selected, and the input consists of RAS values computed with *Expression2RAS* for two datasets: cancer and normal, containing RAS values for tumor and healthy cells, respectively.

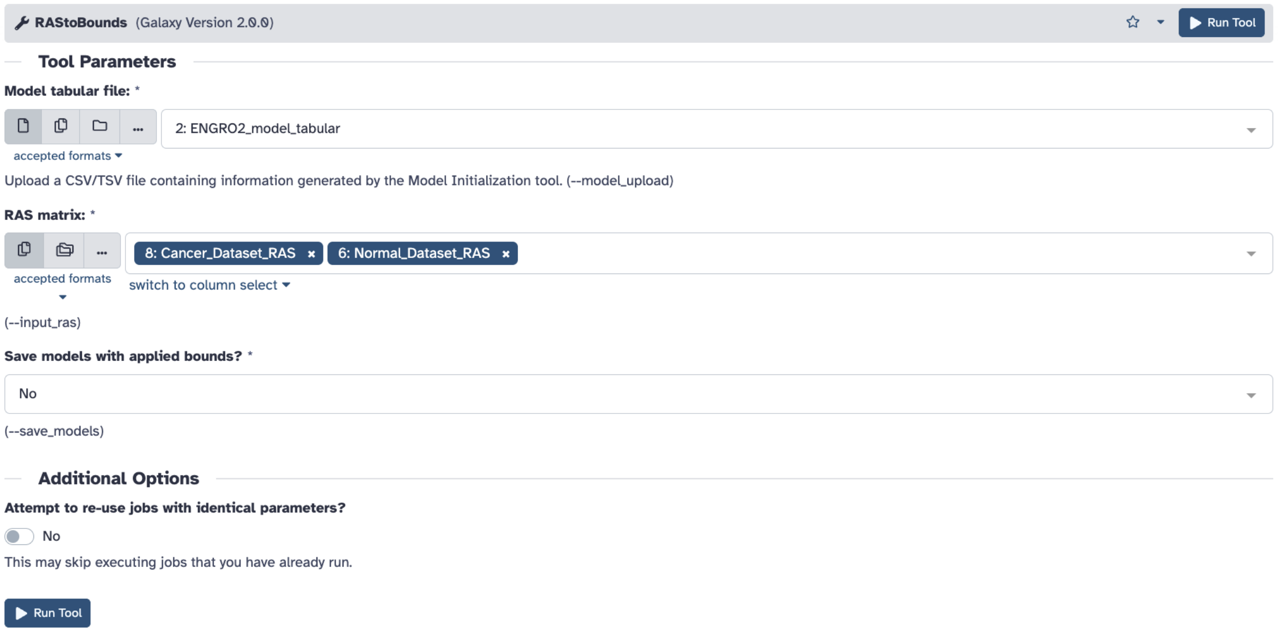

Fig. 4. RAStoBounds tool

Flux Simulation tool

This tool generates flux distributions from a set of metabolic models. Users can provide a single base model and multiple bound files (one per context/cell type), or upload multiple model files, each already containing its own bounds. Two types of analysis are available: flux optimization and flux sampling. For flux sampling, users can specify the sampling algorithm, the number of samples and batches to generate, and the desired outputs (e.g., mean, median, and quartiles). We recommend using 100 samples to reduce execution time.

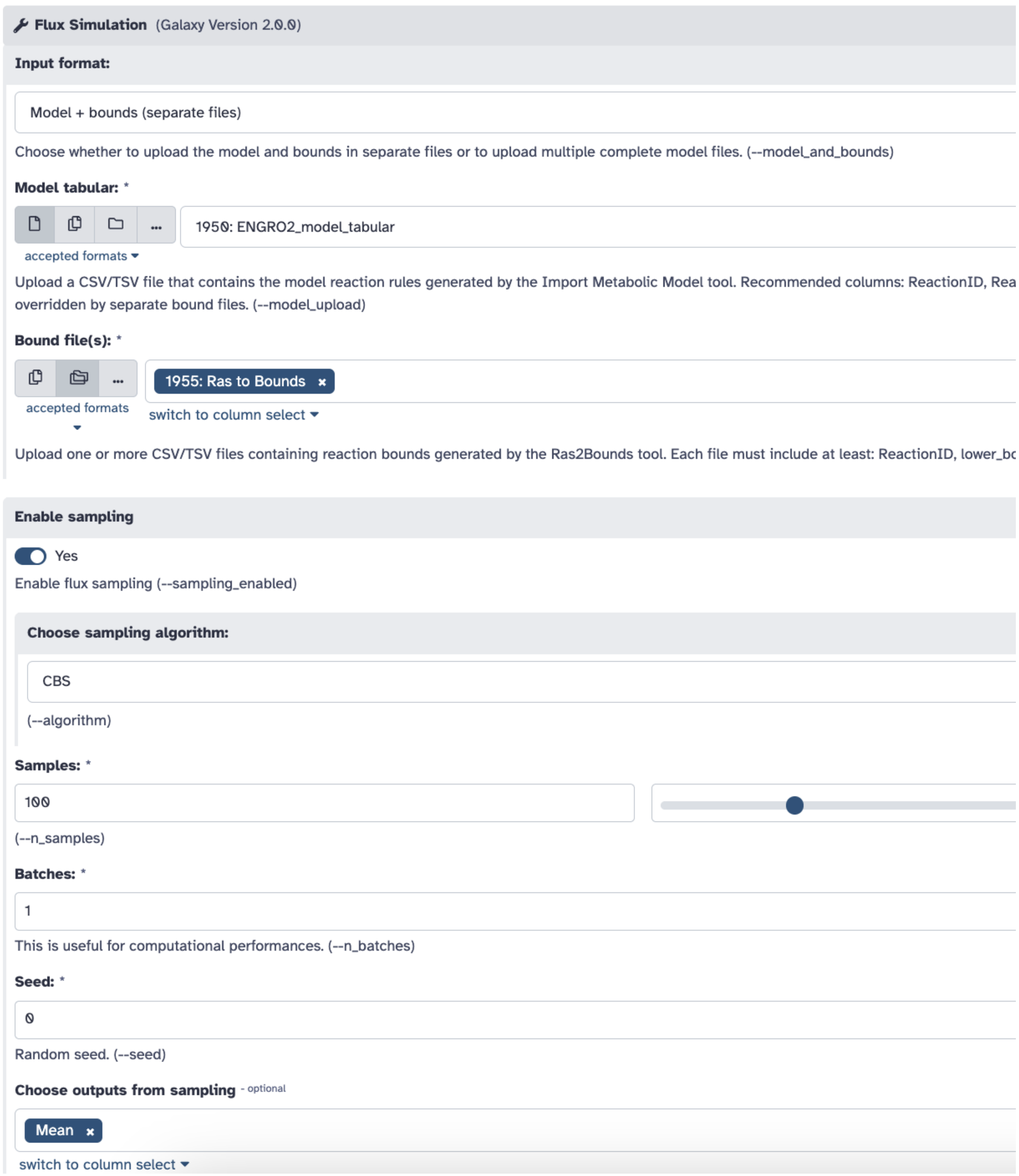

Fig. 5. Flux Simulation tool

Metabolic Flux Enrichment Analysis tool

Finally, users can analyze metabolic differences between different groups using the *Metabolic Flux Enrichment Analysis* tool. To perform the analysis, users need to provide the following inputs: the flux values computed for each sample (in this case, the mean), the class assignments for each sample, the type of comparison (e.g., One vs. One), and the metabolic map of the selected model. This tool identifies statistically significant differences in both the metabolic map and the corresponding tabular output. Specifically, the map is color-coded based on the average flux of each input class, allowing users to easily visualize metabolic differences across conditions.

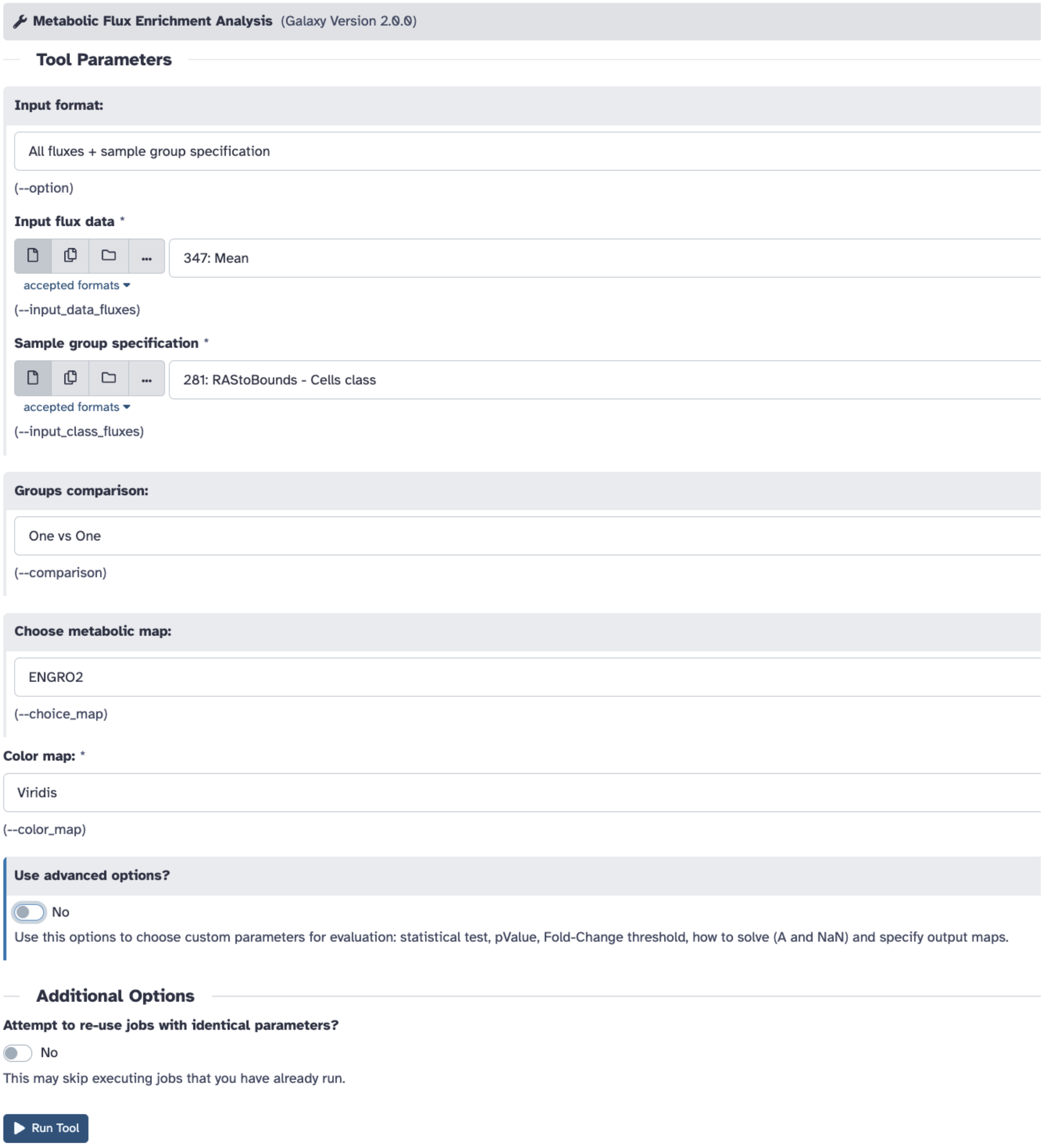

Fig. 6. Metabolic Flux Enrichment Analysis tool

2 Map

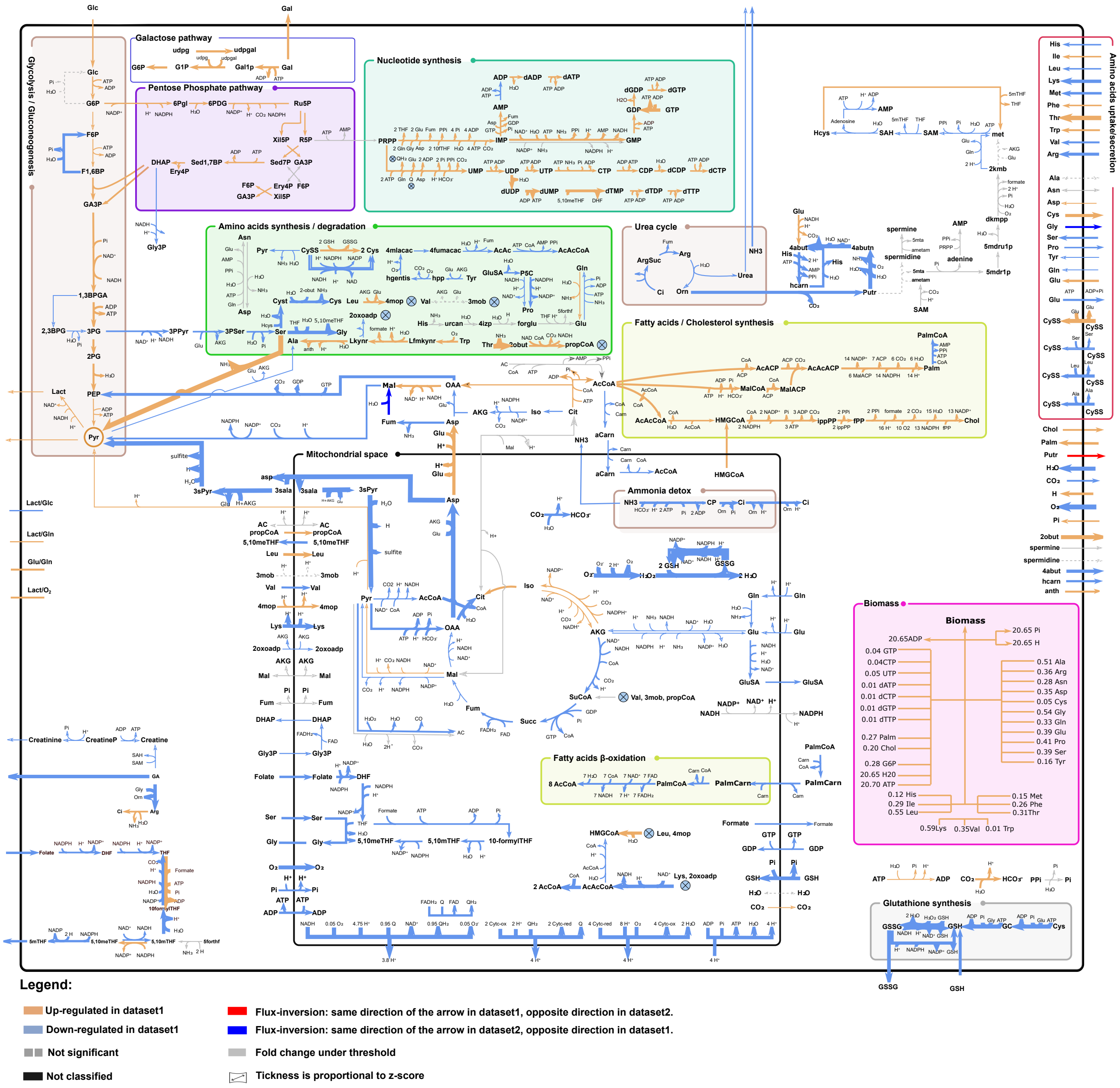

Fig. 1. Comparison of metabolic flux distributions between cancer and normal samples. The figure highlights key metabolic pathways exhibiting significant differences (Kolmogorov–Smirnov test; p-value ≤ 0.05; fold-change $\geq 10\%$) in flux between tumor and healthy conditions, revealing potential metabolic adaptations associated with cancer progression. For this case study, we used the map related to the ENGRO2 metabolic network.
